# Effects of dexamphetamine-induced dopamine release on resting-state network connectivity in recreational amphetamine users and healthy controls

**DOI:** 10.1007/s11682-015-9419-z

**Published:** 2015-07-07

**Authors:** Anouk Schrantee, Bart Ferguson, Diederick Stoffers, Jan Booij, Serge Rombouts, Liesbeth Reneman

**Affiliations:** Department of Radiology, Academic Medical Center, University of Amsterdam, Amsterdam, The Netherlands; Brain Imaging Center, Academic Medical Center, University of Amsterdam, Amsterdam, The Netherlands; Department of Sleep and Cognition, Netherlands Institute for Neuroscience, An Institute of The Royal Netherlands Academy of Arts and Sciences, Amsterdam, The Netherlands; Department of Nuclear Medicine, Academic Medical Center, University of Amsterdam, Amsterdam, The Netherlands; Institute of Psychology, Leiden University, Leiden, The Netherlands; Department of Radiology, Leiden University Medical Center, Leiden, The Netherlands; Leiden Institute for Brain and Cognition, Leiden University Medical Center, Leiden, The Netherlands

**Keywords:** Dopamine, Functional connectivity, Pharmacological MRI, Resting-state fMRI, SPECT

## Abstract

Dexamphetamine (dAMPH) is not only used for the treatment of attention deficit hyperactivity disorder (ADHD), but also as a recreational drug. Acutely, dAMPH induces release of predominantly dopamine (DA) in the striatum, and in the cortex both DA and noradrenaline. Recent animal studies have shown that chronic dAMPH administration can induce changes in the DA system following long-term exposure, as evidenced by reductions in DA transporters, D_2/3_ receptors and endogenous DA levels. However, only a limited number of studies have investigated the effects of dAMPH in the human brain. We used a combination of resting-state functional magnetic resonance imaging (rs-fMRI) and [^123^I]IBZM single-photon emission computed tomography (SPECT) (to assess baseline D_2/3_ receptor binding and DA release) in 15 recreational AMPH users and 20 matched healthy controls to investigate the short-, and long-term effects of AMPH before and after an acute intravenous challenge with dAMPH. We found that acute dAMPH administration reduced functional connectivity in the cortico-striatal-thalamic network. dAMPH-induced DA release, but not DA D_2/3_ receptor binding, was positively associated with connectivity changes in this network. In addition, acute dAMPH reduced connectivity in default mode networks and salience-executive-networks networks in both groups. In contrast to our hypothesis, no significant group differences were found in any of the rs-fMRI networks investigated, possibly due to lack of sensitivity or compensatory mechanisms. Our findings thus support the use of ICA-based resting-state functional connectivity as a tool to investigate acute, but not chronic, alterations induced by dAMPH on dopaminergic processing in the striatum.

## Introduction

Dopamine (DA) neurotransmission plays a key role in regulating motor function, motivation and reward, cognition, and impulsive behavior by modulating oscillations in cortico-striatal-thalamic (CST) networks (Walters et al. 2000). The striatum receives widespread input from the frontal cortex and dopaminergic projections are abundant in this network. DA is thought to modulate cortico-striatal connection strength by increasing the signal-to-noise ratio within these loops (Bamford et al. 2004). Studies using electroencephalography (EEG) and task functional magnetic resonance imaging (fMRI) have also suggested that functional connectivity (FC) in the CST loops could be strongly modulated by dopaminergic neurotransmission (Honey et al. 2003; Williams et al. 2002). Converging evidence reveals that oscillations at the network level can also be assessed with resting-state fMRI (rs-fMRI). For instance, recent animal studies have shown that acute changes in DA concentrations contribute to the regulation of functional connectivity (FC) in the brain. For example, acute administration of the potent psychostimulant dexamphetamine (dAMPH), which increases extracellular DA concentrations by releasing DA and blocking its re-uptake, increased connectivity within the mesolimbic projections of the ventral tegmental area to the ventral forebrain and dorsal thalamic structures of rats (Schwarz et al. 2007). The effect of acute dAMPH on functional connectivity in the CST loops has not been investigated in humans, but acute administration of other dopaminergic stimulant drugs such as methylphenidate (MPH) has been shown to alter CST FC as measured with rs-fMRI (Mueller et al. 2014; Ramaekers et al. 2013; Sripada et al. 2013), as well as in other resting state networks (RSNs), such as the default mode network (DMN) and salience-executive networks (SENs).

Not only the acute effects of dAMPH in humans are poorly understood, studies on the effects of chronic AMPH use on the DA system are also scarce. This is important, since AMPH is used recreationally, and prescribed for treatment of neuropsychiatric disorders such as attention deficit hyperactivity disorder (ADHD) and narcolepsy (Heal et al. 2013). Several animal studies have shown that chronic dAMPH administration induces long-lasting changes to the DA system, such as a reduction in the number of DA transporters (DATs), vesicular monoamine transporters type 2 (VMAT_2_), DA D_2/3_ receptors and DA release (Berman et al. 2009; McCann and Ricaurte 2004; Ricaurte et al. 2005). The few studies that have been conducted in humans reported lower striatal DAT binding and reduced striatal DA release in response to acute dAMPH administration (Schrantee et al. 2014; Reneman et al. 2002) in chronic AMPH users. However, so far, no rs-fMRI studies have been conducted in chronic AMPH users. Nonetheless, a recent study in methamphetamine (METH) users reported increased connectivity in the mesocorticolimbic system in METH dependent subjects when compared with controls (Kohno et al. 2014). More studies have investigated resting-state FC (RS FC) in cocaine-dependent subjects but the results are inconsistent, with both increases and decreases in connectivity having been reported (e.g. Gu et al. 2010; Camchong et al. 2011).

Therefore, the aim of this study was to investigate both the acute and chronic effects of AMPH on resting-state FC in humans, by means of studying recreational AMPH users and healthy controls. Given dAMPH’s strong effects on striatal DA levels, and the findings of previous studies with MPH, we hypothesized that acute dAMPH would modulate CST connectivity and that chronic AMPH users would demonstrate lower change in FC in this network when compared to controls. Because it has been shown that FC within RSNs is strongly associated with DA D_2/3_ receptor expression (Cole et al. 2012; Sambataro et al. 2013), we also investigated whether variations in the individual’s DA neurotransmitter system predict individual RSN FC. [^123^I]IBZM single photon emission computed tomography (SPECT) was used to measure DA D_2/3_ receptor expression and endogenous DA release. [^123^I]IBZM, a selective antagonist for the D_2/3_ receptor, competes with endogenous DA for receptor binding and a DA stimulus such as dAMPH can induce a displacement of [^123^I]IBZM binding that is inversely associated with DA release (Kegeles et al. 1999).

Although we have a strong neurochemical hypothesis to study alterations in the CST network, previous studies have also suggested other networks to be modulated by DA-ergic drugs (Cole et al. 2013). Therefore, we examined the acute and chronic effects of AMPH on the executive, default mode and lateralized fronto-parietal networks in an exploratory analysis.

## Methods

### Participants

Sixteen male AMPH users and age-, gender- and IQ-matched controls were recruited through online advertisements and flyers at local universities and colleges. Inclusion criteria for the AMPH users were at least 30 occasions of use lifetime. The 20 control subjects were healthy volunteers with no self-reported prior use of AMPH or other drugs that affect the dopaminergic system. Exclusion criteria for all participants were a history of a chronic neurological or psychiatric disorder, family history of sudden heart failure, current use of psychoactive medication, abnormal electrocardiogram (ECG), positive drug screen, and a clinical diagnosis of ADHD, in addition to smoking more than 15 cigarettes per day, drinking more than 30 alcoholic beverages per week, and contraindications for undergoing an MRI scan (e.g. ferromagnetic fragments) or the SPECT procedure (e.g. allergy to iodine). The results of the SPECT study in this cohort are more elaborately reported elsewhere (Schrantee et al. 2014). Participants agreed to abstain from smoking, caffeine, alcohol and cannabis for 24 h prior, and for other drugs (including AMPH) one week prior to the assessments. Subjects were asked on the day of the scan if they had used any of these substances in the past two weeks. We verified the reported drug use with a drug screen on a urine sample with an enzyme-multiplied immunoassay for amphetamines, cocaine metabolite, opiates and marijuana). In addition, a detailed drug history questionnaire was obtained and subjects filled in the drug use disorders identification test (DUDIT) to assess substance dependence (Voluse et al. 2012). The Dutch version of the National Adult Reading Test (DART IQ) was conducted as an estimate of verbal intelligence (Schmand et al. 1998).

One participant in the AMPH group showed a positive drug screen and was therefore excluded, so the analyses were performed on 20 controls and 15 AMPH users.

The medical ethics committee of the Academic Medical Center in Amsterdam approved the study procedures and written informed consent was obtained from all subjects and carried out in accordance with the standards of the Declaration of Helsinki.

### Design

**Fig. 1:**
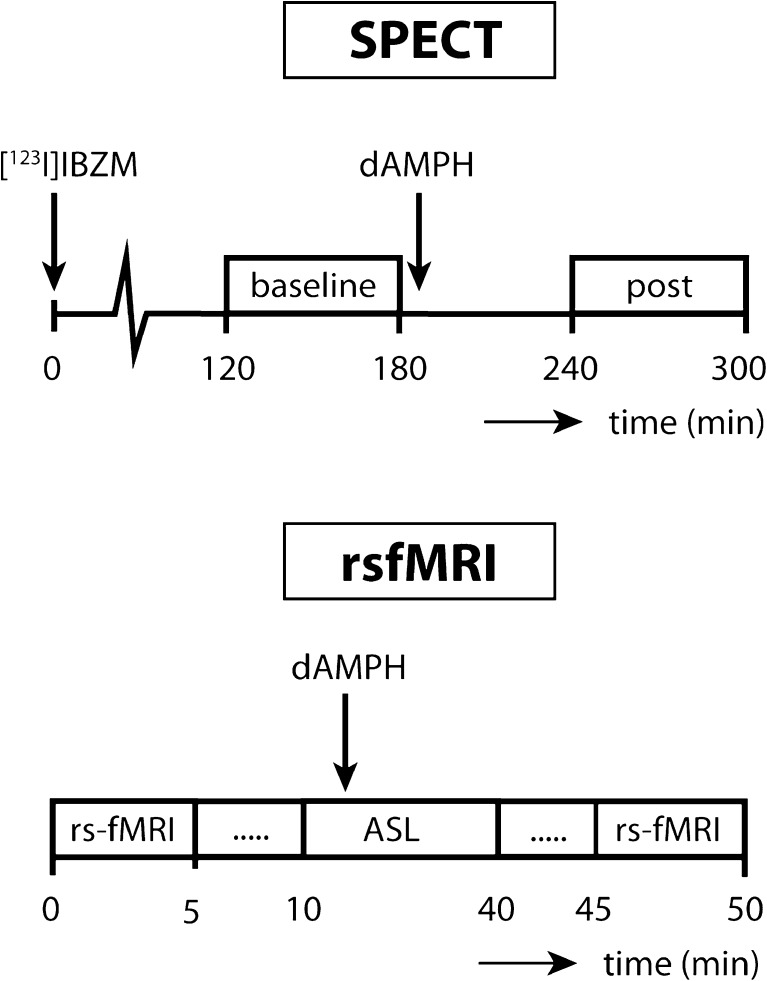
Timeline study protocol

Participants were invited to the hospital for two study visits. On one day they underwent the SPECT scan with dAMPH administration and on the other day they underwent the MRI scan with dAMPH administration. The visits were at least one week apart to allow drug washout and were counterbalanced within groups. Both for the MRI and the SPECT scan, a cannula was inserted in the antecubital vein through which the dAMPH was administered (0.3 mg/kg) during 2 min, followed by 15 ml saline to flush (Fig. 1).

### SPECT acquisition and analysis

Each subject received an intravenous injection of 180 MBq [^123^I]IBZM (80 MBq bolus followed by constant infusion of 20 MBq for 5 consecutive hours). Subjects underwent two [^123^I]IBZM SPECT scans, using the sustained equilibrium/constant infusion technique, to assess baseline striatal DA D_2/3_ receptor availability and the decrease in D_2/3_ receptor binding after the dAMPH challenge, which provides an index of DA release (Kegeles et al. 1999). The radioligand [^123^I]IBZM, which binds with high affinity to DA D_2/3_ receptors, was prepared by GE Healthcare (Eindhoven, The Netherlands). Participants received potassium iodide tablets prior to the SPECT session to block thyroid uptake of free radioactive iodide.

Images were acquired on a brain-dedicated SPECT system (Neurofocus 810, Inc., Medfield, Massachusetts, USA) with the following parameters: matrix: 64 × 64, energy window: 135–190 keV, slice thickness: 5 mm, acquisition time per slice: 300 s, number of slices: 12. SPECT images were corrected for attenuation and reconstructed using iterative algorithms, as earlier described (Booij et al. 1997; Boot et al. 2008). Binding potential (BP_ND_) was calculated as follows: (mean striatal binding – mean binding occipital cortex)/mean binding occipital cortex. Standard templates with fixed ROIs were positioned on the striatum and occipital cortex (reflecting non-specific binding), as earlier described (Booij et al. 1997; Boot et al. 2008; Figee et al. 2014). dAMPH-induced decrease in [^123^I]IBZM BP_ND_, used as a proxy for DA release, was expressed as a % of the pre-dAMPH BP_ND_ (Booij et al. 1997).

### FMRI acquisition

Imaging was performed on a 3-Tesla Ingenia scanner (Philips, Best, The Netherlands) equipped with a 16-channel head coil. A T1-weighted anatomical scan was acquired for registration purposes. For the rs-fMRI scan with blood-oxygenation level-dependent (BOLD) contrast, 130 whole brain volumes of T2*-weighted gradient echo planar images (EPI) were obtained with the following scan parameters: TR/TE 2300/25 ms; flip angle 80°; FOV = 220 × 220 × 117; 39 slices; voxel size 2.3 × 2.3 × 3 mm, SENSE 2.4. The subjects underwent the rs-fMRI scan before dAMPH administration and approximately 30 min post-dAMPH. In the time between the 2 resting-state scans the subjects underwent an arterial spin labeling (ASL) scan. Subjects were instructed to keep their eyes open and let their mind wander.

### FMRI analysis

#### ICA network extraction and connectivity analyses

RS-fMRI data were preprocessed using tools from the FMRIB Software Library (Smith et al. 2004). Brain tissue was extracted for both anatomical and functional images using the brain extraction tool (BET). Functional data were motion corrected, spatially smoothed with a Gaussian kernel of 5 mm FWHM and high-pass temporal filtering at 0.1 Hz was applied. In addition, single session independent component analysis (ICA) was performed to prepare the data for FMRIB’s ICA-based Xnoiseifier (FIX) (Griffanti et al. 2014; Salimi-Khorshidi et al. 2014) to denoise the rs-fMRI data. Prior to analysis, all functional images were first registered to the high-resolution anatomical image (BBR) and subsequently to standard stereotactic space (2 mm, MNI152 template; Montreal Neurological Institute, Montreal QC) using affine transformation. Subsequently, the denoised data were used to investigate RS networks in our groups using multi-session ICA with temporal concatenation (as implemented in FSL MELODIC, Beckmann and Smith 2004). Automatic dimensionality estimation was used to estimate the number of components extracted. In addition to the CST network, we explored 7 networks for further analysis based on their involvement in higher-order cognitive processes and/or DA-ergic function or DA-associated pathologies (Cole et al. 2013). These networks will be referred to as the (i) left-lateralised fronto-parietal network (lFPN) (ii) right-lateralised fronto-parietal network (rFPN) (iii) anterior default mode network (aDMN) (iv) posterior default mode network (pDMN) (v) ventral default mode network (vDMN) (vi) inferior salience/executive network (iSEN) (vii) dorsal salience/executive network (dSEN). For those eight networks, the set of spatial maps from the group-average analysis was used to generate subject-specific versions of the spatial maps, and associated time-series, using dual regression with variance normalization (Beckmann et al. 2009; Filippini et al. 2009). First, for each subject, the group-average set of spatial maps was regressed (as spatial regressors in a multiple regression) into the subject’s 4D space-time dataset. This results in a set of subject-specific time-series, one per group-level spatial map. Next, those time-series were regressed (as temporal regressors, again in a multiple regression) into the same 4D dataset, resulting in a set of subject-specific spatial maps, one per group-level spatial map. To assess baseline group differences, the 4D files for each network were then analyzed using non-parametric permutation testing (5000 permutations) with a two-sample t-test using the FSL Randomise tool (Winkler et al. 2014). To assess the effect of drug and the interaction effect of drug and group, we calculated difference values of the parameter estimates within subjects first using fslmaths, and then performed a one-sample t-test and a two-sample t-test respectively, again using Randomise. All analyses were initially thresholded at *p*-value <0.05 with a family wise error (FWE) correction using threshold free cluster enhancement (TFCE) (Smith and Nichols 2009).

#### Comparison of changes in RS FC and DA release

The 4D files comprising subject-specific spatial maps of voxelwise connectivity with the CST that were obtained from the dual regression analysis, were used to assess the correlation between changes in RS FC and striatal DA release following acute dAMPH administration. We examined specifically this network because it incorporated the striatum, the brain region in which we measured DA release with SPECT, and because dAMPH is thought to exert its greatest effects in the basal ganglia (due to its intense DA-ergic input). A striatal mask was created for the FC map using the MNI structural atlas (Collins et al. 1995). We tested whether individual differences in RS FC within this striatal mask were significantly positively or negatively correlated with DA release as measured with [^123^I]IBZM SPECT within the same striatal mask in the CST using voxelwise non-parametric permutation testing as implemented in Randomise, with 5000 permutations and 5 mm variance smoothing. Voxels were considered significant at *p*-value <0.05 with a FWE correction for multiple comparisons using TFCE.

#### Effects of heart rate of RS FC

Changes in heart rate (HR) have been found to influence the BOLD signal, especially near large vessels, and consequentially influence FC in RSNs (Khalili-Mahani et al. 2013). Dopaminergic drugs such as dAMPH are known to increase HR and blood pressure (Lile et al. 2011). Although FIX denoises the data and thereby removes a lot of physiological variation, it has been shown that areas of physiological variation can overlap with our RSNs of interest. Therefore, we decided to repeat our analyses using average HR as a covariate. HR was monitored during the MRI scan session using a 4-lead vector cardiogram (VCG) signal and data were extracted using an in house MATLAB script. Five of our HR measurements were missing and they were imputed with the average HR of the group and session the scan belonged to.

## Results

### Demographics

Demographics and AMPH use are provided in Table 1. There were no significant differences in the age (t_(33)_ = 0.04, *p* = 0.97) and IQ (t_(33)_ = −0.07, *p* = 0.95) between both groups. In addition to AMPH, the user group also contained more smokers and cannabis users and used significantly more alcohol (U = 214.50, *p* = 0.03), MDMA (U = 300.00, *p* < 0.01) and cocaine (U = 280.00, *p* < 0.01) than our control group. The highest score on the DUDIT amongst AMPH users was 16, well below the cut-off score of 25 of probable drug dependence (Voluse et al. 2012). The AMPH users typically used AMPH as a powder, with the main routes of administration being inhalation or by dissolving it in a drink.

**Table 1:** Group characteristics

	Healthy controls (*N* = 20)	AMPH users (*N* = 15)	*p*-value
Demographics			
Age (y)^1^	21.10 ± 2.77	21.07 ± 1.49	0.97
IQ^1^	104.10 ± 8.49	104.27 ± 5.02	0.95
Drug use			
Amphetamine use lifetime (g)^2^	–	18 ± 28	NA
occasions	–	40 ± 60	NA
average dose (g)	–	0.25 ± 0.3	NA
age of onset (y)	–	18 ± 2	NA
MDMA use (occasions)^2^	0 ± 0	6 ± 1	<0.01
Cocaine use (occasions)^2^	–	2 ± 3	<0.01
Nicotine use (number of smokers)	0	9	NA
Nicotine use (cig/week)^2^	–	10 ± 2	<0.01
Cannabis use (number of users)	1	9	NA
Alcohol use (units/week)^2^	6.75 ± 14	14 ± 17	0.03

^1^values represent mean ± standard deviations with Student’s t-test

^2^values represent median ± interquartile range with Mann–Whitney U-test

### Effect of acute dAMPH administration on RS FC patterns

**Fig. 2:**
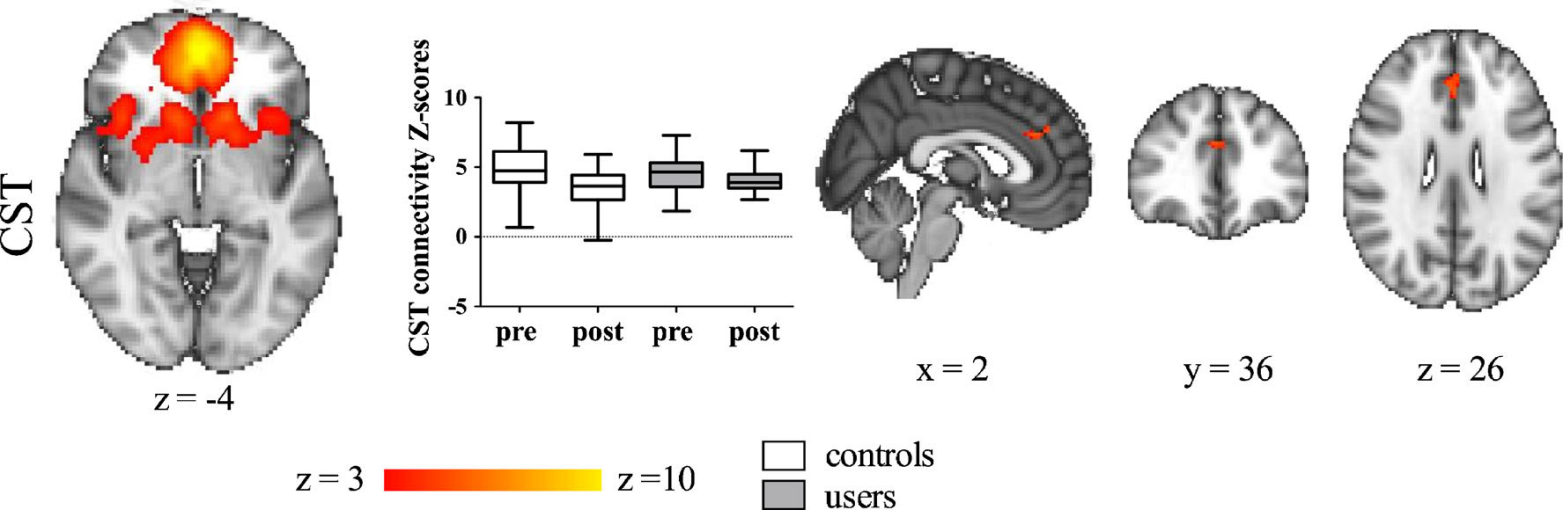
dAMPH induces network-specific changes in FC in CST. dAMPH induces network-specific changes in CST network FC. Left: Axial slices of RSN maps extracted with ICA. Middle: Z-scores of connectivity with RSN for the largest cluster for both controls (white) and users (grey), pre- and post-dAMPH administration. Both users and controls display a decrease in connectivity with the RSN following dAMPH. There was no significant interaction between group and dAMPH administration on RSN connectivity. Right: Brain regions displaying significant dAMPH-induced reductions in connectivity with the RSN, for all subjects (*P* < 0.05, FWE corrected). Slices in all figures displayed in radiological convention; coordinates provided in MNI standard space

**Fig. 3:**
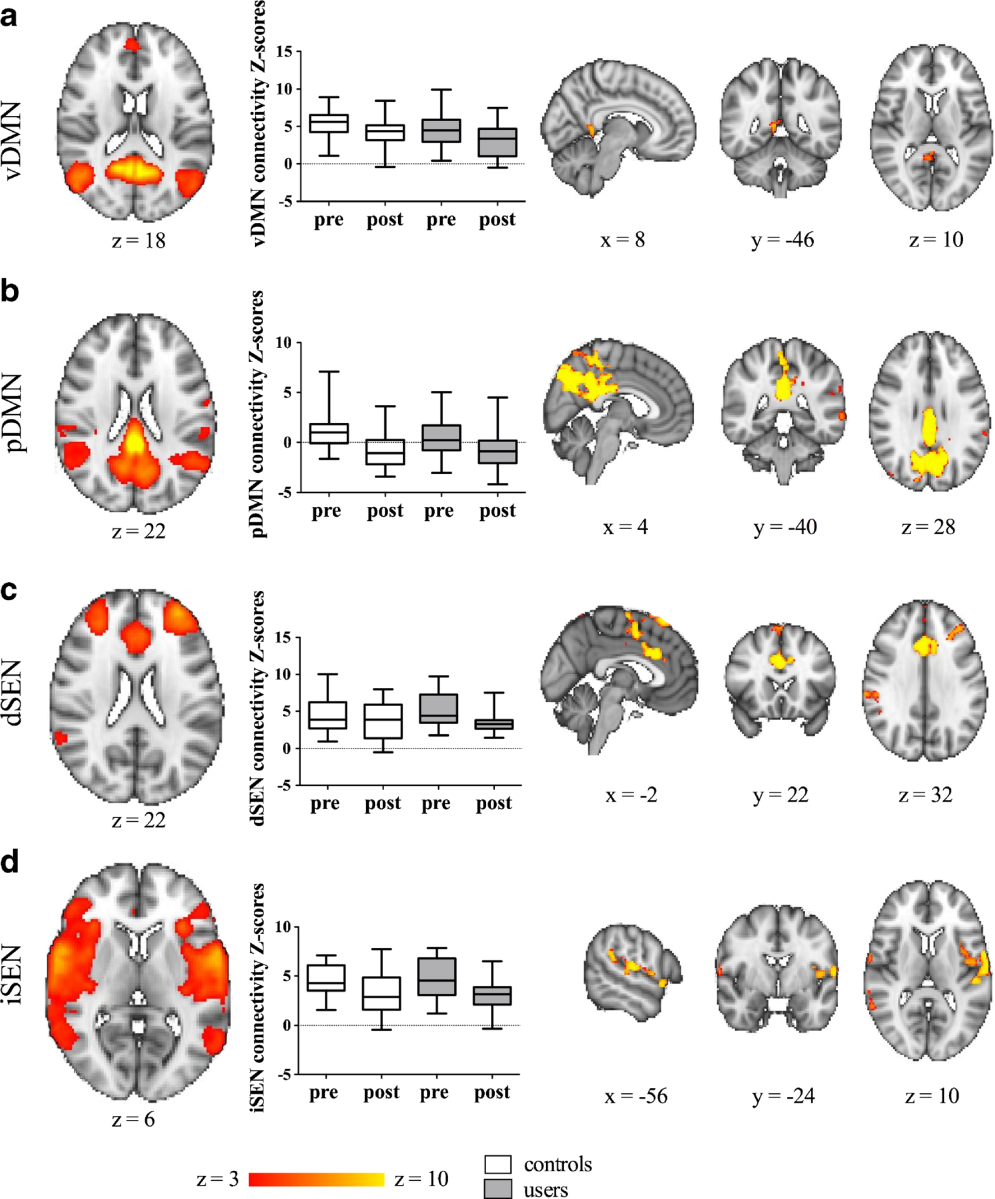
Exploratory analyses of dAMPH induces network-specific changes in FC. dAMPH induces network-specific changes in resting-state connectivity. A-D, left: Axial slices of RSN maps extracted with ICA. A-D, middle: Z-scores of connectivity with RSN for the largest cluster for both controls (white) and users (grey), pre- and post-dAMPH administration. Both users and controls display a decrease in connectivity with the RSN following dAMPH. There was no significant interaction between group and dAMPH administration on RSN connectivity. A-D, right: Brain regions displaying significant dAMPH-induced reductions in connectivity with the RSN, for all subjects (*P* < 0.05, FWE corrected). Slices in all figures displayed in radiological convention; coordinates provided in MNI standard space

We found a significant main effect of dAMPH challenge on FC patterns in a small cluster in the ACC with the CST network (Fig. 2; Table 2). In addition, in exploratory analyses, multiple monoaminergic networks showed reduced FC following dAMPH administration (Fig. 3; Table 2). The dSEN showed decreased connectivity to the anterior cingulate cortex and left middle frontal gyrus, bilaterally, after dAMPH administration. On the other hand, the iSEN showed decreases in the left precentral gyrus. Additionally, pDMN (and to a lesser extent vDMN) showed very strong reductions in connectivity, mostly with the posterior cingulate node of this network. No significantly different FC patterns were found within the lFPN, rFPN and aDMN.

**Table 2:** Brain regions displaying significant decreases of FC following dAMPH, relative to baseline

RSN of interest	Brain region	Voxels	Max t	MNI coordinates		
				*x*	*y*	*z*
CST	BL paracingulate gyrus, anterior cingulate gyrus	49	4.71	0	40	28
vDMN	R posterior cingulate gyrus	68	5.53	8	−48	6
pDMN	BL precuneous cortex, posterior cingulate cortex	4945	8.35	−2	−70	34
	R central opercular cortex	150	4.88	40	−70	48
	L middle temporal gyrus	123	4.09	−45	−58	8
	L planum temporale	107	4.48	−64	−26	16
	L middle temporal gyrus	95	4.43	−66	−44	0
	L supramarginal gyrus, anterior	44	3.42	−62	−36	36
dSEN	BL anterior cingulate cortex	1387	6.51	2	20	30
	L middle frontal gyrus	342	6.2	−32	38	44
	R supramarginal gyrus, anterior	149	4.71	64	−28	32
iSEN	L precentral gyrus	982	5.49	−62	−10	10
	R temporal pole	168	5.05	52	12	−4
	L middle temporal gyrus	92	4.63	60	−52	8
	R supramarginal gyrus	51	5.27	−62	−26	42

CST, cortico-striatal-thalamic network; dSEN, dorsal salience executive network; iSEN, inferior salience executive network; pDMN, posterior default mode network; vDMN, ventral default mode network; clusters >40 voxels are reported

We found a trend significant increase in HR during the resting state scan after dAMPH administration (t(33) = −1.80, *p* = 0.08). We did not find any significant changes between the analyses with HR as a covariate when compared to the analyses without covariate.

### Effect of chronic dAMPH on RS FC patterns

The recreational AMPH users did not differ significantly from the control group in their resting-state FC patterns, nor in their response to an acute dAMPH challenge. In Fig. 2 and Fig. 3 the differences in Z-scores of both groups before and after dAMPH are shown separately.

### RS FC associated with striatal D_2/3_ receptor availability and DA release

**Fig. 4:**
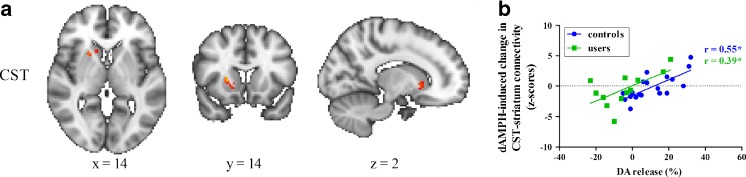
Effect of DA release on changes in CST connectivity with the striatum. (A) dAMPH-induced changes in functional connectivity are positively associated with dAMPH-induced DA release in the striatum (*P* < 0.05, FWE corrected). Slices are displayed in radiological convention; coordinates provided in MNI standard space. (B) Both users and controls display this positive correlation and there is no significant difference between the slopes

We previously reported reductions in DA release in the current cohort of AMPH users compared to controls (Schrantee et al. 2014). In line with this, acute dAMPH-induced changes in resting-state FC between the CST network and the striatum were positively associated with extent of striatal DA release (Fig. 4) in both controls and AMPH users. Although the correlation coefficient between DA release and CST FC resting-state connectivity did not differ between AMPH users and controls (F(1,28) = 0.01, *p* = 0.91), in AMPH users it was displaced to the left, indicating more negative or blunted responses to dAMPH in this group. In AMPH users CST FC correctly identified blunted DA release in nearly all cases, but in more than half of the control subjects with a positive DA release (Fig. 4). Post-hoc, we also determined whether striatal change was negatively associated with change in ACC RS FC, but this correlation was only apparent at a trend level (*r* = −0.25, *p* = 0.07). As expected, we did not find a correlation between DA release and baseline RS FC. We also did not observe any correlation between baseline striatal DA D_2/3_ receptor availability and baseline striatal connectivity with the CST network either.

## Discussion

The present study demonstrates that acute dAMPH administration reduced connectivity within the CST network and that striatal DA release, but not baseline striatal DA D_2/3_ receptor availability, was associated with changes in striatal resting-state connectivity in this network. In addition to the CST, large effects of acute dAMPH administration were observed in the salience executive networks and default mode networks. Contrary to our hypothesis, we did not observe a group difference in dAMPH-induced changes in RSNs.

The cortex, striatum and thalamus are connected by means of large-scale parallel loops with direct monosynaptic connections from the cortex to the striatum and indirect, polysynaptic connections from the striatum, through the thalamus and back to the cortex (Shepherd 2013). This wiring is thought to be crucial for its role in cognition and motor function and DA has been shown to modulate these connections (Cole et al. 2013; Honey et al. 2003). Indeed, we found a small cluster in the ACC that showed reduced connectivity with the CST network following dAMPH administration. This is in accordance with other studies, showing that drugs that increase DA release or elevate DA levels (such as MPH and L-DOPA respectively) decrease FC within regions of the CST network. For instance, L-DOPA administration has also been found to reduce connectivity of the ACC with a CST network (Cole et al. 2013). Honey et al. (2003), demonstrated that MPH, a DAT and noradrenaline transporter blocker, reduced connectivity between the caudate nucleus and the midbrain, without showing an effect on the connectivity between the caudate nucleus and the thalamus. Similarly, Mueller et al. (2014) reported a decrease in cortical and subcortical components of the CST network following MPH. In addition, Ramaekers et al. (2013) found reduced connectivity following MPH administration between the ventral and dorsal striatum, as well as between the ventral striatum and the medial prefrontal cortex (mPFC). Konova et al. (2013) also focused on the CST network but found opposite effects, with MPH decreasing intra-striatal connectivity, but increasing cortico-striatal connectivity. However, this was in a sample of cocaine dependent subjects, which has been shown to have altered connectivity patterns (Konova et al. 2013).

We also demonstrated that dAMPH-induced striatal DA release was associated with changes in CST resting-state FC in the striatum following dAMPH administration. This is in line with animal studies in which microdialysis measurements of striatal DA release are associated with BOLD and CBF changes following dAMPH administration (Chen et al. 1997). Our correlational analysis was performed for the striatum, because this is the brain region where we also measured DA release, whereas differences in RS FC following dAMPH administration were measured for the whole brain. The neuronal effects of dAMPH are thought to originate mainly from the basal ganglia, as dAMPH increases extracellular DA primarily in these regions (Chen et al. 1997). Yet, in our study, we did not find any subcortical areas that showed altered connectivity following dAMPH administration. However, dopaminergic and noradrenergic (NA) terminals are also widely distributed in cortical areas (Lewis et al. 1987). Indeed, a recent microdialysis study showed that administration of dAMPH increases both DA and NA in the prefrontal cortex, but only DA in the striatum (Rowley et al. 2014). As such, modulation of RSNs in the striatum may be mainly determined by DA release, whereas cortical RS FC is both dependent on changes of DA and NA. This could also explain why we find a decrease in ACC connectivity, despite increases in striatal DA release. This is further supported by the inverted-U hypothesis of DAergic modulation, suggesting that there is an optimal level of DAergic stimulation, with both too little and too much DA negatively impacting behavior. Indeed, previous studies have already shown that DA modulations can induce inverted-U relationships in cortical FC patterns (Cole et al. 2013). Therefore, it is conceivable that FC decreases in the cortex when DA release is acutely increased to a large extent in subcortical areas. In this context, it would be very interesting to study dose–response relationships of DA modulation on RS FC.

Contrary to what we expected, no significant group differences nor any interaction effects on RS FC patterns were found, even though the groups under study differed markedly on baseline striatal DA D_2/3_ receptor availability and dAMPH-induced DA release, as we reported elsewhere (Schrantee et al. 2014). This is possibly due to the sensitivity of rs-fMRI measurements to pick up regional DA alterations. SPECT is a direct method to investigate DAergic receptor binding, whereas rs-fMRI is an indirect tool to assess temporal coherent activity between brain regions and could therefore be less sensitive to small DA alterations in part of a network. This is in contrast with studies reporting alterations in CST connectivity in subjects with other drugs of abuse acting on the DA system, such as cocaine and METH (Gu et al. 2010; Kohno et al. 2014; Konova et al. 2013). The largest difference between those studies and the present one is that those subjects suffered from substance dependence, whereas our subjects were typically recreational users. It is extremely difficult to disentangle the effects of addiction per se and neurotoxicity of the drug that subjects are addicted to. Yet, it is possible that addiction in itself induces changes in resting state connectivity that are not evident in recreational users of a drug. For example, neurobiological correlates of intoxication differ from those of craving and compulsory drug administration (Goldstein and Volkow 2002; Koob and Volkow 2010). A related explanation might be that although our users displayed alterations in DA release, compensatory changes in the CST loops may have ‘buffered’ alterations in resting state connectivity. Such compensatory measures may be sufficient to maintain normal CST connectivity in case of recreational use, but might fail when subjects become addicted to a drug. Although no studies have directly compared RS FC between recreational use and addiction, the transition phase from abuse to addiction has been associated with compensatory mechanisms (Kalivas and Volkow 2005).

In addition to acute changes in the CST network, we found reduced connectivity in DMNs and SENs following dAMPH administration. This is concordance with other studies that also found changes in connectivity after DA drug administration in networks outside the CST loops. In our study, the most prominent effect of acute dAMPH on FC was in the DMN, a network in which connectivity is suppressed during task performance. We found reductions of connectivity primarily in posterior parietal regions. Failure to inhibit DMN activity during task performance is associated with worse behavioral performance (Weissman et al. 2006). Our findings are in concordance with a study by Kelly et al. (2009) that showed that L-DOPA, a DA precursor, decreased DMN activity, and Nagano-Saito et al. (2008) that demonstrated that DA depletion increased DMN activity in healthy controls. In addition to changes in DMN connectivity we also found reductions in connectivity within salience-executive networks (dSEN and iSEN), including mostly frontal areas. Salience-executive networks are thought to play a role in reward and cognitive processing (Seeley et al. 2007). These findings are in line with task fMRI literature showing that administration of oral dAMPH reduces activation in a monetary incentive delay task (Knutson et al. 2004). However, Cole et al. (2013) reported no changes in SEN networks following L-DOPA administration, whereas Mueller et al. (2014) found increased connectivity of SENs with the visual cortex, but decreased connectivity with the cerebellum and thalamus. Although we found large reductions of RS FC in DMN and SEN’s, these analyses were exploratory in nature and therefore we cannot draw strong conclusions from them. Still, the large effects observed in these networks, highlight the importance of whole brain analyses for rs-fMRI data. Networks in the brain are not single entities, but can influence other networks and work in close concordance. It would be interesting for further research to assess the effects of DA drugs on between-network connectivity (Smith et al. 2013).

This study investigated the effects of dAMPH administration on resting-state BOLD signal fluctuations. The BOLD signal is influenced by a large number of factors including CBF, cerebral blood volume, and oxygenation. dAMPH has been shown to be a vasoconstrictive agent systemically and in the brain by DA receptors on microvessels (Choi et al. 2006). Therefore, we cannot exclude vascular contributions (unrelated to neuronal effects) to the acute dAMPH-induced changes in RS FC as reported here. In addition, in this study we administered the drug intravenously rather than orally, as most studies so far have done. Intravenous administration induces a large strain on the cardiovascular system which may influence the BOLD response (Heal et al. 2013). However, we observed that the large physiological effects are primarily present directly following administration and have largely subsided at the time of our rs-fMRI sequence 30 min later (Heal et al. 2013). In addition, adding HR as a covariate in our statistical analysis did not affect our results. Therefore, it is unlikely that cardiovascular changes contributed to the acute dAMPH-induced RSN modulations. In addition, we used FIX to remove residual physiological variation and motion (Griffanti et al. 2014). So, the potential confounding cardiovascular effects were reduced to a minimum in this study. In addition, in between resting-state scans subjects underwent a resting-state ASL scan, which means that there were no confounding effects of cognitive tasks that could influence subsequent resting-state FC (Gordon et al. 2014).

In this study, the participants received a dAMPH challenge twice, which could potentially impose an order effect. To account for this, we counterbalanced the visits within groups, with half of the subjects first undergoing the SPECT scans and the other half first undergoing the MRI scan. One more limitation of this study is that subjects always underwent the dAMPH scan after the baseline scan, which could induce expectancy effects (Gundersen et al. 2008). However, subjects were aware that they would not yet receive the dAMPH challenge until the ASL scan 15 min later. In addition, if the anticipation differed between AMPH users and controls, one would expect AMPH users to have more anticipation of reward and therefore more [^123^I]IBZM displacement than the controls. Yet, we observed a blunted response in the AMPH users compared to controls. Nevertheless, future studies could assess the expectancy effects by including a placebo condition. In addition, an inherent limitation to studies investigating recreational drug use is polydrug use. Indeed, in our sample, AMPH users also used significantly more other drugs and we can therefore not exclude that our results are due to other drug use. However, the subjects underwent a drug screening prior to the scans, which excludes acute effects of other drugs of abuse on our results. In addition, the effect of acute administration on the CST network is consistent with studies investigating the effect of MPH and L-DOPA on CST FC. However, polydrug use could have increased variation within the dAMPH user group and this could have resulted in a lower power to detect group differences. In addition, many different analysis techniques are employed, but comparisons between for example ICA and seed-based approaches have not found significant differences in reproducibility and ability to detect resting-state networks (Franco et al. 2013; Hale et al. 2015). In fact, for inter-individual differences ICA-based approaches may be more sensitive than seed-based approaches (Smith et al. 2014), which is reflected in the association we observed between striatal DA release and RS FC. Yet, it is possible that other techniques are more sensitive than ICA-based techniques to detect group differences in specific subcortical DA-ergic regions, but this postulate remains to be tested. Another potential limitation is that our resting-state scan contained 130 volumes, which is a relatively short scan. However, studies have shown that resting state scans of 5 up to 13 min provide reliable estimates of resting state connectivity networks (Birn et al. 2013). Although our scan of 5 min falls within that time window, it is possible that longer resting-state scans (up to 12 min) provide more information and could thus be more sensitive to detect the difference between our recreational dAMPH users and controls. This would be an interesting topic for further research.

## Conclusion

In conclusion, we demonstrate that acute dAMPH reduces RS FC within multiple RSNs and that individual variation in DA release following an acute challenge with dAMPH, predicts changes in resting-state FC. These results suggest an important role for both DA (and NA) in the modulation of RS FC by dAMPH. However, we did not find significant group differences in RS FC, possibly due to lack of sensitivity or compensatory mechanisms for reduced DA release.
